# Diagnosing Pediatric Patients With Hereditary C1-Inhibitor Deficiency—Experience From the Hungarian Angioedema Center of Reference and Excellence

**DOI:** 10.3389/falgy.2022.860355

**Published:** 2022-05-04

**Authors:** Noémi Andrási, Zsuzsanna Balla, Beáta Visy, Ágnes Szilágyi, Dorottya Csuka, Lilian Varga, Henriette Farkas

**Affiliations:** ^1^Department of Internal Medicine and Haematology, Hungarian Angioedema Center of Reference and Excellence, Semmelweis University, Budapest, Hungary; ^2^School of Ph.D. Studies, Semmelweis University, Budapest, Hungary; ^3^2nd Department of Pediatrics, Semmelweis University, Budapest, Hungary; ^4^Department of Infectious Diseases, Heim Pál Children's Hospital, Budapest, Hungary; ^5^Research Laboratory, Department of Internal Medicine and Haematology, Semmelweis University, Budapest, Hungary

**Keywords:** hereditary C1-inhibitor deficiency, diagnosis, pediatric, umbilical cord blood, complement and genetic testing

## Abstract

**Background:**

Hereditary Angioedema with C1-inhibitor deficiency (C1-INH-HAE) is a rare disease characterized by recurrent subcutaneous and/or submucosal edematous (HAE) episodes, which may occur at any age. The mean age of the symptom onset is 10–12 years. Diagnostic protocols differ by age group and family history.

**Methods:**

We retrospectively analyzed clinical and laboratory data (C4-, C1-INH concentration and function) from 49 pediatric patients diagnosed with C1-INH deficiency at our Angioedema Center between 2001 and 2020. Moreover, we analyzed the connection between complement parameters and symptom onset.

**Results:**

From the 49 pediatric patients [boy/girl: 23/26, the average age of diagnosis: 6.7 years (min: 0-max: 18.84)], the majority (36/49, 73%) was diagnosed as the result of family screening. Of all the enrolled patients, 34% (17/49) experienced symptoms before the diagnosis. During the observational period, 33% (16/49) of the patients remained asymptomatic, while 33% (16/49) became symptomatic. The average age at symptom onset was 7.8 years (min: 0.5–max: 18). Only 27% (13/49) of pediatric patients were diagnosed after referrals to our center because of typical symptoms. From those patients diagnosed with family screening, 4/36 experienced symptoms at or before the time of the diagnosis. In the case of five newborns from the family screening group, umbilical cord blood samples were used for complement testing. In the case of 3/36 patients, the first complement parameters did not clearly support the disease, but the presence of the mutation identified in the family verified the diagnosis. Complement results were available from 11 patients who became symptomatic during the observational period. Complement parameters 1 year prior to and after the onset of symptoms were compared, and significantly lower concentrations of C1-INH (*p* = 0.0078) were detected after the onset of symptoms compared to the preceding (symptom-free) period.

**Discussion:**

The majority of pediatric patients were diagnosed as a result of family screening before the onset of symptoms. Early diagnosis allows supplying the patients with special acute treatment for HAE attacks, which may occur at any time. Our results highlight the importance of DNA analysis in pediatric patients in case of a known mutation in the family, and an ambiguous result of complement testing.

## Introduction

Hereditary angioedema developing with C1-INH deficiency (C1-INH-HAE) is a rare autosomal dominantly inherited disease, where the estimated prevalence is 1:50,000. It is characterized by recurrent subcutaneous and/or submucosal edematous episodes. The clinical appearance of this disease is diverse: from completely asymptomatic patients to patients experiencing angioedematous attacks on a monthly basis or even more frequently. Symptoms can occur at any age, but, in half of the patients, the first symptom occurs before the age of 18 years (1, 2). The average time of the appearance of symptoms is 10–12 years (3). Since this is a rare disease, health care practitioners tend not to recognize it; therefore, the diagnosis is often late, and almost half of the patients have at least one misdiagnosis in their medical history prior to the correct diagnosis. In childhood, angioedema occurring on the gastrointestinal submucosa is one of the most common symptoms, which may mimic the clinical picture of acute abdomen. A rare but life-threatening manifestation of the disease is angioedema occurring in the upper airways, which, in childhood, may cause suffocation in a short time due to the smaller airway diameter of children (1, 3). These factors significantly increase disease burden with frequent emergency department admission, unnecessary therapies, and surgical procedures, moreover, raise the risk of death from upper-airway obstruction (1, 4). Bradykinin has a role in the pathomechanism of C1-INH-HAE. This mediator is responsible for vasodilation and increased vascular permeability, which causes the development of edema. Due to this, HAE attacks do not respond to conventional antihistamines, glucocorticosteroids, or adrenaline used in histamine-mediated angioedema. Instead, C1-INH-HAE requires special treatment, which is targeted to supplement the missing protein, inhibit the formation of bradykinin, or inhibit bradykinin's effect on bradykinin 2 receptors (2, 5).

In 2017, an international consensus was published on the diagnosis and care of C1-inhibitor-deficient individuals under the age of 19 years (3). In pediatric patients, diagnosis is based on complement measurement, similar to that in adults. Low C1-inhibitor and C4 concentration, besides a low C1-inhibitor function, support the diagnosis of the first type of the disease (C1-INH-HAE type I). If the C1-INH concentration is normal or even increased but the functional activity of the protein is decreased and the level of C4 is low, then the second type of the disease is suspected (C1-INH-HAE type 2). In newborns, the level of C4 and C1-INH may be decreased because the complement system is not matured at this age. Therefore, to establish a diagnosis based on complement testing, at least two congruent complement results verifying C1-INH deficiency are necessary, out of which the second test needs to be done after the child has turned 1 year old. If the mutation causing the disease in the family is known and genetic testing is available, the analysis of the *SERPING1* gene encoding the C1-inhibitor protein may provide an opportunity for the diagnosis, especially under the age of 1 year, when ambiguous complement results may occur. In the case of newborns, it is possible to perform complement and genetic testing from umbilical cord blood (UCB), and if there is a family member diagnosed with C1-INH-HAE, until the exclusion/confirmation of the disease, C1-inhibitor deficiency should be presumed, even in asymptomatic cases (3, 6, 7).

This study aimed to assess the process of diagnosing C1-INH deficiency in children under 19 years (pediatric patients) in the Hungarian Angioedema Center of Reference and Excellence from approximately the last 20 years. We examined the advantages of the applied methods (complement measurement and genetic testing) in clinical practice and the time of appearance of clinical symptoms and their connection to complement parameters.

## Materials and Methods

We retrospectively analyzed clinical and laboratory data from 49 Hungarian patients who were diagnosed with hereditary C1-inhibitor deficiency during their childhood (<19 years of age). Clinical data, such as the reason for specialized medical investigation (family screening or typical symptoms), age at diagnosis, and symptom appearance, together with laboratory data (complement parameters, identified genetic variation), were collected from the National Database of Hereditary Angioedema. All the patients included in the study were diagnosed, managed, and followed up regularly at the Hungarian Angioedema Center of Reference and Excellence between 2001 and 2020. The study protocol was approved by the institutional review board of the Semmelweis University of Budapest, and informed consent was obtained from the participants in accordance with the Declaration of Helsinki.

Complement testing was performed in all cases. Two types of samples were used for complement testing: peripheral blood and umbilical cord blood (if it was available). The C1-inhibitor activity was measured with a commercially available ELISA kit (Quidel, San Diego, US), while radial immunodiffusion was used to assess C1-inhibitor concentration. The C4 complement levels were detected by turbidimetry (Cobas Integra 400 analyzer; Roche, Switzerland).

If the C1-INH functional level was lower than the normal range together with a low C4 level, and the patient had low C1-INH concentration, the diagnosis of HAE with C1-INH deficiency type I was established. Patients with low C1-INH function, along with a normal or elevated C1-INH concentration, were diagnosed as having C1-INH-HAE type II. All symptom-free newborns or infants with a parent or sibling with C1-INH-HAE were considered to have C1-INH deficiency until this diagnosis was excluded, and these patients were supplied with special emergency treatments.

Analysis of the *SERPING1* gene was performed in each case. Isolated DNA samples were analyzed by bidirectional Sanger sequencing following PCR amplification, and the presence of copy-number alterations (deletions or duplications) in the *SERPING1* gene was detected by using multiplex ligation-dependent probe amplification (MLPA), applying the SALSA MLPA P243 SERPING1 kit (MRC-Holland, Amsterdam, The Netherlands).

Moreover, we analyzed the connection between complement parameters (C4-, C1-INH concentration and function) and the symptom onset. The patients who became symptomatic during the follow-up period and had complement results in the preceding 1 year and after symptom appearance were included. Besides, C1-INH concentration was compared in 8 pediatric patients who were asymptomatic and had complement parameters in the year after the diagnosis. Paired sample *t*-tests were used for statistical analyses. The level of statistical significance was set at 0.05.

## Results

Of the 49 pediatric patients, 23 were boys and 26 were girls. The average age at the time of diagnosis was 6.7 years (min: 0-max.: 18.84, median: 5). The average follow-up period in our center was 8.83 years (min.: 0.21- max.: 19.42, median: 7.9). Two out of 49 patients had C1-INH-HAE type 2; the other 47 patients had C1-INH-HAE type 1. Regarding the starting point of the diagnosis, the majority of the pediatric patients (36/49, 73%) were diagnosed as the result of family screening. The other group of patients (13/49, 27%) was diagnosed after referrals to our center because of typical symptoms. In the case of 2/13 patients, the disease was not present in any available family members. While, in 11/13 cases, family screening following the diagnosis verified the presence of further affected family members.

The average age at symptom onset was 7.8 years (min.: 0.5–max.: 18, median: 6.5). Of all the enrolled patients, 34% (17/49) experienced symptoms before the diagnosis. During the follow-up period, 33% (16/49) of the patients stayed asymptomatic, while 33% (16/49) became symptomatic. From the 36/49 group (the family screening group), only 4/36 experienced symptoms at or before the time of the diagnosis. In the case of symptomatic patients, the diagnostic delay was 3.5 years on average (min.: 0–max.: 11.7, median: 3).

Of all the enrolled pediatric patients, 11 were newborns (all term babies) or infants when they were diagnosed (<1 year of age). In the case of five newborns, UCB samples were used; in the other six cases, peripheral blood serum was tested to establish the diagnosis. In all five cases where UCB samples were analyzed, the complement parameters clearly confirmed the diagnosis: in 4 cases, typical results for C1-INH-HAE type 1, and, in 1 case, typical findings for C1-INH-HAE type 2 were detected. In the remaining 6 infants, peripheral blood serum was tested, and complement measurements provided uncertain results in 2 cases; in both cases, C1-INH function was in a normal range, with low levels of C4 and C1-INH in one case (Patient 1), while, in the other case (Patient 2), C1-INH concentration was also in a normal range. In these two cases, genetic analysis verified the presence of a pathogenic *SERPING1* variation. In the case of Patient 1, the C1-INH function stayed in the normal range in repeated measures (in a yearly checkup), and no HAE attack occurred during the observational period. In the case of Patient 2, C1-INH-HAE type 2 was diagnosed, and, later on, (when he was older than 1 year of age), low functional activity of C1-INH was detected. Patient 2 had his first HAE attack at the age of 5. One other child (more than 1 year of age at the time of diagnosis) had normal complement results; C1-INH function, -concentration, and C4 concentration were in a normal range; however, the known *SERPING1* mutation in the family could be detected with genetic testing. In this patient, a year after the diagnosis C1-INH function, - concentration were found to be low; however, C4 levels still stayed in the normal range.

**Figure 1:**
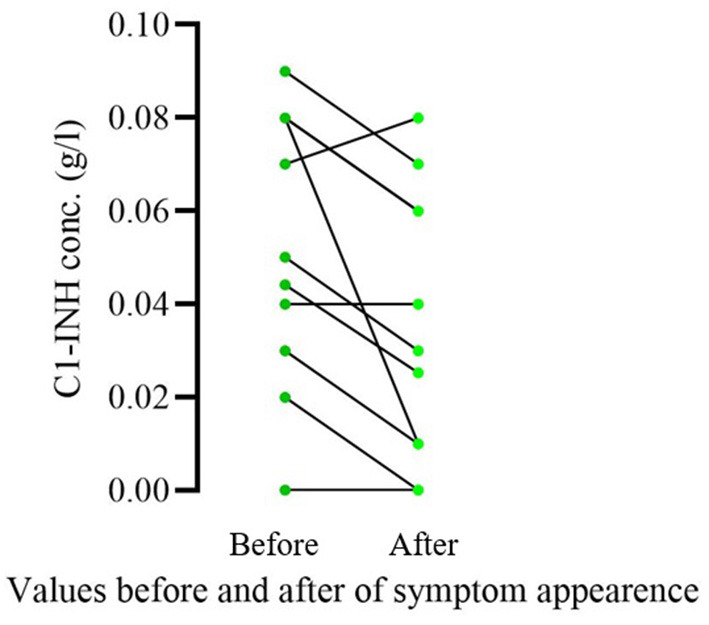
Comparison of C1-INH concentration prior vs. after the onset of symptoms in pediatric patients diagnosed as a result of family screening. Only patients who became symptomatic during the follow-up period and had complement results in the 1 year before and after symptom appearance were included in this analysis (11/16). Significantly lower C1-INH concentrations were detected (*p* = 0.0078) after the onset of symptoms in these patients.

**Figure 2:**
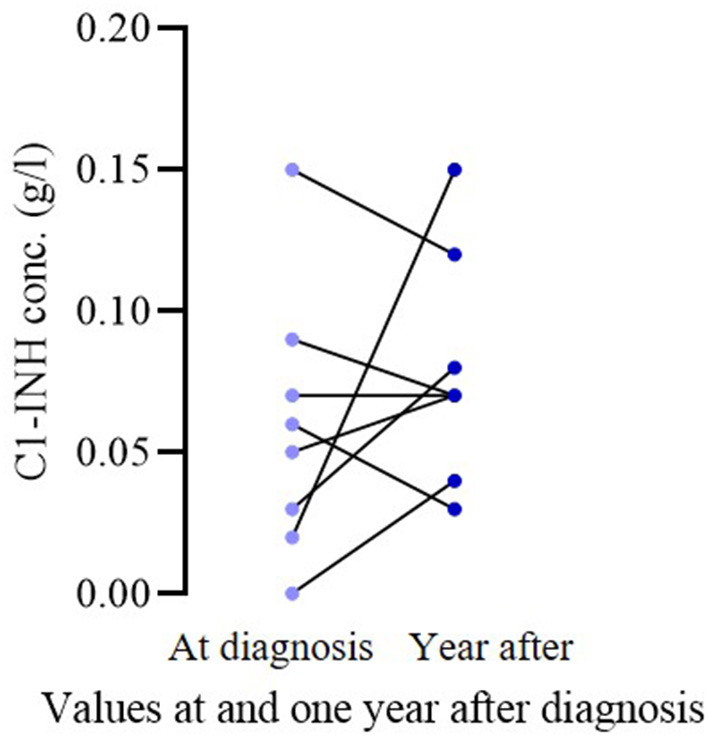
Comparison of C1-INH concentration at diagnosis vs. a year after in asymptomatic pediatric patient. In the case of 8 asymptomatic patients, the C1-INH levels at diagnosis and in the year after were compared. No significant change could be detected (*p* = 0.4219).

Of the 16 patients who became symptomatic, 11 had been sampled in the 1 year before and after symptom appearance. We compared complement parameters measured in samples obtained prior to and after the occurrence of symptoms. The C1-INH concentrations were significantly lower (before occurrence median: 0.05; IQ range: 0.05; after occurrence median: 0.03; IQ range: 0.05; *p* = 0.0078) after the onset of symptoms in these patients (Figure 1). In the case of C4 and C1-INH functions, no significant changes could be detected. Sixteen patients remained asymptomatic through the follow-up period; 8 of them were analyzed at diagnosis and a year after as well. In these patients, no significant change was found in C1-INH concentration in samples collected before and 1 year after diagnosis (at diagnosis median: 0.055; IQ range: 0.0625; after diagnosis median: 0.07; IQ range: 0.0625; *p* = 0.4219) (Figure 2).

## Discussion

Here, we summarized our experience of the past 20 years in diagnosing children with C1-INH-HAE. Processing the clinical and laboratory data of pediatric patients diagnosed with C1-INH deficiency in our Angioedema Center between 2001 and 2020, we found that 65% of the pediatric patients were diagnosed as a result of family screening without preceding edematous episodes. The mean age at the first appearance of the symptoms in the case of our patients was 7.8 years, which is lower than the average 10 years found in the literature. It is known that there may be significant differences between different populations in the appearance of symptoms, so, for example, in the case of Chinese patients, the first symptoms typically occur at the age of 20–30 years (3, 8). The median delay in the diagnosis was 3 years in our study, which is considerably lower than the value described in the literature (median of 8.5 years) (9). During the surveillance period, 33% of our patients remained asymptomatic; a similar percentage of asymptomatic children was found in an Italian survey as well (10).

Based on previous literature data, the concentration of C1-INH, its function, and the level of C4 may be lower than the applied reference range (determined by analyzing healthy adults) even in healthy children under the age of 1 year, which may cause a diagnostic difficulty in some cases (6, 11, 12). However, in a study by Pedrosa et al. published in 2016, only the level of C4 seemed not to be reliable in this age group. They studied nine pediatric patients under the age of 1; out of whom, four turned out to have C1-INH deficiency. In all four cases, the C1-INH function and concentration were low, while, in the five children who did not have C1-INH deficiency, these values were in the normal range. However, in 4/5 cases in the non-deficient group, the value of C4 was under the lower reference limit (7). Analyzing complement results in our cohort revealed that, in the case of 2/11 patients under the age of 1 year, the first complement test showed normal C1-INH function, but, since the mutation of the *SERPING1* gene was known in the family, the affected *SERPING1* exon was analyzed, and the presence of the disease-causing mutation was verified. Moreover, there was one child in the age group older than 1 year, in case of whom the first complement results were also normal, but, since the mutation of the *SERPING1* gene was known in the family, molecular genetic testing was applied that confirmed the diagnosis of C1-INH-HAE. These results highlight the importance of DNA analysis in pediatric patients with positive family history and a known mutation in the family.

In a paper by Nielsen et al. in 1994, C1-INH deficiency was proved with complement testing performed from UCB samples (6). There are no further published data analyzing the reliability of UCB samples for the diagnosis of C1-INH deficiency. In our study, the diagnosis of five newborns was established based on the complement parameters measured from UCB; all five samples had unambiguous complement results, verifying the presence of the disease, which was confirmed by genetic testing.

In the case of 11 patients whose symptoms developed during the follow-up period and had a complement result in 1 year, preceding the appearance of the first symptoms, we found that the concentration of C1-INH was significantly lower in the blood samples taken after the appearance of HAE symptoms compared to the values measured prior to the appearance of the symptoms. A similar decrease could not be observed as regards the C1-INH concentration of asymptomatic patients, when comparing their levels at the diagnosis and 1 year later, excluding the possibility of an age-related decrease in symptomatic patients. These results suggest that further decrease of C1-INH levels may favor the appearance of HAE symptoms or HAE symptoms are accompanied by a decrease in C1-INH concentration. This is in agreement with previous results, showing a connection between the C1-INH level and the initiation of HAE attacks (13, 14).

We aimed to study and evaluate the diagnostic process of the patients with pediatric C1-INH deficiency followed up in the Hungarian Angioedema Center of Reference and Excellence. Our results emphasize the importance of family screening and newborn screening in the establishment of an early diagnosis, ideally prior to the appearance of symptoms, as this provides an opportunity to offer adequate treatment modalities before unnecessary hospitalization and medical procedures happen. Although we have limited data, our few cases suggest that the earliest diagnosis can be established safely from UCB samples. It should be highlighted that, in case of a positive family history, when the *SERPING1* mutation is known, genetic testing is a safely applicable and recommended method.

## Data Availability Statement

The datasets generated for this study are available on request to the corresponding author.

## Author Contributions

NA: research topic development, data collection, and the wording of the article. HF: research topic development, article creation, and review of the article. DC and ÁS: genetic analyses. LV: complement measurements. ÁS, ZB, BV, DC, and LV: article creation and review. The final version of the article was read and approved by all the contributors. All authors contributed to the article and approved the submitted version.

## Funding

This study was supported by NKFI 124557.

## Conflict of Interest

The authors declare that the research was conducted in the absence of any commercial or financial relationships that could be construed as a potential conflict of interest.

## Publisher's Note

All claims expressed in this article are solely those of the authors and do not necessarily represent those of their affiliated organizations, or those of the publisher, the editors and the reviewers. Any product that may be evaluated in this article, or claim that may be made by its manufacturer, is not guaranteed or endorsed by the publisher.

## Abbreviations

C1-INH: C1-inhibitor
C1-INH-HAE: hereditary angioedema with C1-inhibitor deficiency
DNA: deoxyribonucleic acid
ELISA: enzyme-linked immunoassay
HAE: hereditary angioedema
MLPA: multiplex ligation-dependent probe amplification
PCR: polymerase chain reaction
UCB: umbilical cord blood.
